# Seasonal changes in the diversity, host preferences and infectivity of mosquitoes in two arbovirus-endemic regions of Costa Rica

**DOI:** 10.1186/s13071-022-05579-y

**Published:** 2023-01-26

**Authors:** Luis M. Romero-Vega, Marta Piche-Ovares, Claudio Soto-Garita, Daniel Felipe Barantes Murillo, Luis Guillermo Chaverri, Alejandro Alfaro-Alarcón, Eugenia Corrales-Aguilar, Adriana Troyo

**Affiliations:** 1 Universidad de Costa Rica, San José, Costa Rica; 2 Universidad Nacional, Heredia, Costa Rica; 3 Auburn University, Auburn, Alabama USA; 4 Universidad Estatal a Distancia, San José, Costa Rica

**Keywords:** Mosquito, Diversity, NDVI, Alphavirus, Flavivirus

## Abstract

**Background:**

Mosquitoes are vectors of various arboviruses belonging to the genera* Alphavirus* and* Flavivirus*, and Costa Rica is endemic to several of them. The aim of this study was to describe and analyze the community structure of such vectors in Costa Rica.

**Methods:**

Sampling was performed in two different coastal locations of Costa Rica with evidence of arboviral activity during rainy and dry seasons. Encephalitis vector surveillance traps, CDC female gravid traps and ovitraps were used. Detection of several arboviruses by Pan-Alpha and Pan-Flavi PCR was attempted. Blood meals were also identified. The Normalized Difference Vegetation Index (NDVI) was estimated for each area during the rainy and dry seasons. The Chao2 values for abundance and Shannon index for species diversity were also estimated.

**Results:**

A total of 1802 adult mosquitoes belonging to 55 species were captured, among which *Culex quinquefasciatus* was the most caught species. The differences in NDVI were higher between seasons and between regions, yielding lower Chao-Sørensen similarity index values. Venezuelan equine encephalitis virus, West Nile virus and Madariaga virus were not detected at all, and dengue virus and Zika virus were detected in two separate *Cx. quinquefasciatus* specimens. The primary blood-meal sources were chickens (60%) and humans (27.5%). Both sampled areas were found to have different seasonal dynamics and population turnover, as reflected in the Chao2 species richness estimation values and Shannon diversity index.

**Conclusion:**

Seasonal patterns in mosquito community dynamics in coastal areas of Costa Rica have strong differences despite a geographical proximity. The NDVI influences mosquito diversity at the regional scale more than at the local scale. However, year-long continuous sampling is required to better understand local dynamics.

**Graphical Abstract:**

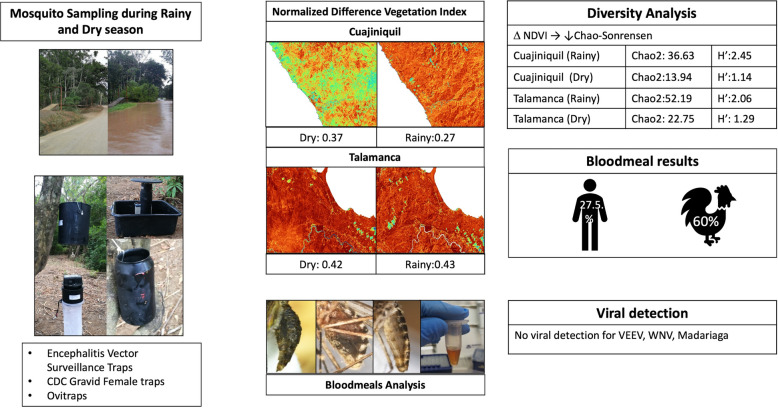

**Supplementary Information:**

The online version contains supplementary material available at 10.1186/s13071-022-05579-y.

## Background

Mosquitoes are vectors of several arboviruses belonging to the genus *Alphavirus* (e.g. Venezuelan equine encephalitis virus (VEEV] and Mayaro virus) and genus* Flavivirus* (e.g. West Nile virus [WNV], Zika virus (ZIKV) and dengue virus [DENV]) [1]. In some cases, enzootic transmission cycles occur between vertebrate animals and mosquitoes, with potential transmission of the arbovirus to humans [2–4]. Vector diversity can affect transmission patterns by amplifying or reducing disease risk [5]. Moreover, emerging or re-emerging arboviruses need competent vectors to favor the propagule pressure (number of introduction attempts and individuals) and establish themselves in new environments. These competent vectors often form complex communities with other mosquito species. It has been hypothesized that fluctuations between competent and non-competent mosquito species can influence arboviral transmission dynamics. Less competent vectors can maintain viral transmission when the abundance of the primary vector has decreased [5–7]. These dynamics can be harder to address and study in highly diverse mosquito communities where less competent vectors can trigger epidemics or buffer a potential dilution effect [8].

Vertebrate host diversity and species richness have also been established as significant predictors for vector-borne disease transmission risk [8, 9]. Depending on the pathogen and host communities, this correlation can be positive or negative. Mosquito community assemblage is also know to affect virus survival in terms of long-term transmission [6]. Since most empirical work focuses on primary vector species, these diversity traits have not been deeply explored for disease vectors. Furthermore, most epidemiological models assume homogeneity in a vector community [5].

Research on the effect of vector diversity on disease prevalence is scarce. One study has found that vector richness can increase the prevalence of malaria [10], but empirical data obtained on multiple vectors for the WNV did not show any effect on disease prevalence [5], making this hypothesis still elusive.

Costa Rica is among those countries in the world with the highest number of mosquito species per area unit [11]. Consequently, this high species richness can assemble complex mosquito communities within which several arboviruses can maintain enzootic transmission cycles. Several arboviruses with enzootic transmission involving wildlife hosts, including VEEV [12, 13], Madariaga virus (MADV; part of the eastern equine encephalitis virus [EEEV] complex that circulates in Central and South America) [14, 15], WNV and Saint Louis encephalitis virus (SLEV), are endemic in Costa Rica and have been detected in horses and other wild and domestic animals [16, 17]. However, only a limited number of studies have attempted to characterize the community of vectors in Costa Rican endemic areas, and these are outdated. One of the most comprehensive nationwide studies was conducted in 1940 as part of the United Fruit Company’s efforts to control malaria in their workers, resulting in 24,704 mosquitoes from 93 species being collected [18]. During the epidemics that occurred in the 1970s, VEEV was isolated from *Deinocerites pseudes* and from *Aedes taeniorhynchus* collected with CDC light traps [13]. During an outbreak of yellow fever in 1953, *Haemagogus spegazzinii* was identified as the possible vector [19]. More recently, mosquito blood-feeding patterns from several species were described at Lomas Barbudal, a wetland near Cuajiniquil (CU) in Costa Rica [20]*.* More recent mosquito research in Costa Rica has focused chiefly on the primary vectors of dengue (DENV), ZIKV and chikungunya (CHIKV) viruses, namely *Aedes aegypti* and *Aedes albopictus* [21–23], due to their importance in public health.

In contrast with the neighboring country of Panama and other countries of Latin America, mosquito vector communities in arbovirus endemic areas of Costa Rica are mostly unknown [24–26]. Therefore, this research aimed to characterize and compare the mosquito vector and non-vector community structure in two endemic areas for arboviruses in Costa Rica during a rainy and dry season, as well as to identify blood-meal sources to establish the feeding patterns of putative vectors in the areas. Finally, we tried detecting possible arboviruses currently circulating in these mosquito communities.

## Methods

### Study site

The study was performed in two coastal locations with evidence of arboviral activity in Costa Rica: Cuajiniquil (CU) and Talamanca (TA) [27]. CU is a district with patches of secondary tropical dry forest located on the northwest coast of Costa Rica. Its main economic activities are cattle ranches and tourism. TA is a county on the southeast coast with patches of old growth and secondary tropical rainforest. Its current main economic activities are banana plantations and cattle ranches mixed within forest patches. CU (average annual precipitation of 1800 mm across 97 rainy days) has a severe dry season, with almost no precipitation, from December to April and a rainy season from May to November [28]. TA (yearly average precipitation of 3710 mm across 193 rainy days) has a long rainy season from November to July with a decrease in rainfall from August to October, but the dry season is not as severe as that of CU [28]. A total of eight sampling points per location were selected, subsequently subdivided into four different collection settings: domiciliary (DO), peridomiciliary (PE), animal pen (PN) and forest (FO). Criteria for each sampling point were: (i) they had to be inhabited; (ii) horses and chickens had to be present; (iii) there had to be a FO area that was at least 50 m distant from the DO area.

### Mosquito sampling and initial processing

Mosquitos were caught in CU from May to December 2017 (rainy season) and from January to April 2018 (dry season); in TA, trapping was carried out from May to July 2018 (rainy season) and from August to October 2018 (dry season). Three different mosquito trapping methods were used per sampling location: four encephalitis vector surveillance (EVS) traps (Bioquip Products Inc., Compton, CA, USA), three CDC female gravid traps (GTs) (John W Hock Co., Gainesville, FL, USA), and three ovitraps (OVs) [29]. EVS traps were baited with CO^2^ at a 300 l/min flux using the CO_2_ tank adapter (BIoquip Products Inc.), and GT were baited with a hay infusion. EVS traps and GTs were set between 18:00 h and 6:00 h for one night per season at each sampling location; the OVs were left at each study site for 2 weeks. The EVS trap was the only trap placed at all four sampling settings (DO, PE, PN, and FO); GTs and OVs were not set at DO. A manual collection of larvae in natural breeding sites was also performed in each locality to enable a better description of the species composition. All visible larval habitats were sampled, including artificial containers, phytotelmata, ponds and puddles. Depending on the water volume, each container was tested using a turkey baster or a D-Frame Water Net (Bioquip Products Inc.). Larvae were sorted in a larval tray (Bioquip Products Inc.), and all visualized individuals were deposited in 70% ethanol.

The adult mosquitoes collected were freeze-killed and transported to the Vectors Research Laboratory (LIVe) at the University of Costa Rica to be identified at the species level. Larvae (fixed in 70% ethanol) were later mounted on glass slides using a polyvinyl alcohol mounting medium (Bioquip Products Inc.). All specimens were identified using the* Key for the Mosquitoes of Costa Rica* [30]. Adults caught in EVS traps at all sampling points were pooled by sex, species (1 pool of up to 20 individuals per species) and collecting zone. Females caught in GTs were placed individually in 1.5-ml microcentrifuge tubes (Eppendorf, Hamburg, Germany). Adults caught in EVS traps and GT adults were stored in RNAlater (Thermo Fisher Scientific, Waltham, MA, USA) until nucleic acid extractions. In mosquito pools from EVS traps, only RNA was extracted using TRIzol reagent (Invitrogen, Thermo Fisher Scientific), according to the manufacturer’s instructions, followed immediately by complementary DNA (cDNA) synthesis by random primers using RevertAid™ (Thermo Fisher Scientific). For individual mosquitoes captured in GTs, RNA and DNA were extracted using NucleoSpin™ TriPrep Columns (Macherey–Nagel GmbH, Düren, Germany); RNA was processed as described above. All DNA and cDNA were quantified using a NanoDrop™ spectrophotometer (Thermo Fisher Scientific) and stored at −20 °C until molecular amplification.

### Viral detection

Pan-PCRs were used to detect captured species of * Alphavirus* and* Flavivirus* molecularly. For *Flavivirus*, a semi-nested PCR was used to amplify a 220-bp fragment of the NSP4 gene using primers cFD2 (5ʹ-GTGTCCCAGCCGGCGGTGTCATCAGC-3ʹ) and MAMD (5ʹ-CATGATGGGRAARAGRGARRAG-3ʹ) for the first reaction, and primers cFD2 and FS778 (5ʹ-AARGGHAGYMCDGCHATHTGGT-3ʹ) For the second reaction [31]. PCR amplifications were carried out in a total volume of 25 µl (12.5 µl of GreenTaq Master Mix [Thermo Fisher Scientific], 1 µl of each primer [10 nM], 7.5 µl of water [Fermentas, Thermo Fisher Scientific] and 3 µl of cDNA). Cycling conditions for the first PCR were: 1 cycle for 5 min at 95 °C; followed by 25 cycles of 1 min at 95 °C, 1 min at 53 °C and 1 min at 72 °C; with a final extension at for 7 min at 72 °C. For the second PCR, the cycling conditions were: one cycle of 5 min at 94 °C; followed by 35 cycles of 1 min at 94 °C, 1 min at 54 °C and 1 min at 72 °C; with a final extension for 7 min at 72 °C. cDNA of the DENV-1 Angola genotype was used as a positive control for all reactions with *Flavivirus*.

For* Alphavirus*, a nested PCR protocol was applied to amplify a 210-bp product using the primers PanAlphaOutF (5’-TTTAAGTTTGGTGCGATGATGAAGTC-3ʹ) and PanAlphaOutR (5ʹ-GCATCTATGATATTGACTTCCATGTT-3ʹ) for the first reaction and PanAlphaInF (5ʹ-GGTGCGATGATGAAGTCTGGGATGT-3ʹ) and PanAlphaInR (5ʹ-CTATGATATTGACTTCCATGTTCATCCA-3ʹ) for the second reaction [32]. Cycling conditions for the first PCR were: 1 cycle of 15 min at 45 °C; 1 cycle of 3 min at 95 °C; 10 cycles of 20 s at 95 °C, 1 min at 55 °C and 1.5 min at 72 °C; with a final extension of 7 min at 72 °C. For the second PCR, the cycling conditions were: 1 cycle of 2 min at 92 °C and 40 cycles of 20 s at 95 °C, 20 s at 58 °C and 20 s at 72 °C, as previously described [32]. The PCR mixture was the same as described above. For a positive reaction control, cDNA from VEEV strain TC-83 was included.

All amplicons were visualized by electrophoresis in 1.5% agarose gel (Bio-Rad Laboratories, Hercules, CA, USA) stained with GelRed™ (Biotium Inc., Fremont, CA, USA) and using Borate-EDTA 0.5× (Thermo Fisher Scientific) as a medium buffer. Amplicons were compared with a 100-bp molecular ruler (Thermo Fisher Scientific) with its corresponding positive control size. Positive amplicons were purified using ExoSap IT™ (Thermo Fisher Scientific) and sequenced (sense and antisense) elsewhere (Macrogen®, Seoul, South Korea).

### Blood-meal detection

Only blood-fed females collected with GTs were used for blood-meal detection. Blood meals were scored according to the BF1-3 classification, and any individual with evidence of blood-feeding within the last 72 h was included in the analysis [33]. A 244-bp product was amplified by single-step PCR using the primers ModRepCOIF (5ʹ-TNTTYTCMACYAACCACAAAGA-3ʹ) and VertCOI7216R (5ʹ-CARAAGCTYATGTRTTYATDCG-3ʹ); the PCR cycling conditions were: one cycle of 3 min at 94 °C; followed by 40 cycles of 40 s at 94 °C, 30 s at 48.5 °C and 1 min at 72 °C; with a final extension of 7 min at 72 °C, as previously described [33]. DNA from an *Ae. aegypti* (Rockefeller strain) blood-fed on *Mus musculus* (BALB/c) was used as a positive reaction control. The PCR reaction mix, amplicon visualization and sequencing steps were performed as described above.

### Sequence identification

Viral and blood-meal consensus sequences were generated and aligned using the MEGA X™ software. Each consensus sequence was identified according to its closest percent similarity with reference sequences using the BLAST® tool. We considered a percentage of similarity > 98% for virus sequences as a correct identification [33]. For vertebrate blood-meal sequences, the sequence with the highest percentage of similarity (> 90%) and lowest E-value was considered to be the most suitable match.

### Data analyses

For diversity analyses, EstimateS™ software 9.1 version was used [34]. According to the software user's guide, data were loaded using the “Type 1” format. Species richness was estimated for the EVS traps with the Chao2 index [35] using 100 replicates and the “not corrected” method, according to software recommendations, due to an incidence distribution > 0.5. Diversity was estimated using the Shannon index for species diversity. The Shannon index describes the homogeneity (evenness) of the community while the Chao2 index extrapolates presence/absence values. The Shannon index was estimated not only for the values from the EVS traps as a total but also per season (rainy and dry), sampling site (CU and TA) and sampling area (DO, PD, PE, and FO). Evenness (*E* = *e* ^*∧*^ *H* ^'^/*S*) was also estimated using the Shannon diversity index [36]. The Chao-Sørensen similarity index was used to assess the similarity in species composition among sampling areas (DO, PD, PE, and FO) between seasons and locations. The Chao-Sørensen similarity index ranges from 1 to 0, where lower values indicate a higher dissimilarity in species composition [37]. In addition, species accumulation curves were created using rarefaction and extrapolation with Hill numbers, using the R package iNext [38].

### Normalized Difference Vegetation Index and Normalized Difference Water Index

The Normalized Difference Vegetation Index (NDVI) and the Normalized Difference Water Index (NDWI) for a local and regional scale was calculated to correlate seasonal fluctuations (absolute difference) with the Chao-Sørensen similarity index. The NDVI and NDWI values were calculated in QGIS with Landsat-8 satellite imagery obtained from LandsatLook™ (US Geological Survey, Reston, VA, USA). Each NDVI and NDWI was calculated for each county during each season. The selection criteria for the image were: (i) it had to be taken during each sampling season; (ii) there had to be a maximum of 25% cloud coverage; and (iii) the buffer area for the sampling point in each image (radius: 2 km) must not have any cloud coverage. Because of the reduced number of available images, a time series reflecting changes in the NDVI and NDWI was impossible. Therefore, we only could make a comparison between seasons. Any value between - 1 and 0 was filtered to extract any data coming from the ocean or due to heavy cloud cover [39]. For the computation of the local and regional NDVI and NDWI, we established a buffer area with a 2-km radius (local scale) for each sampling point, which was based on the general foraging capacity of mosquitoes (mainly *Culex*) on edges of fields and forest [40]. Each 2-km area was considered for the local NDVI and NDWI values. The regional NDVI and NDWI values were obtained by calculating the mean of all local NDVI or NDWI per county per season. To assess if variation in the NDVI and NDWI affected the turnover of mosquito populations between seasons in each county, we did a Pearson’s correlation between the NDVI and NDWI variation (rainy season NDVI and NDWI vs. dry season NDVI and NDWI) and the Chao-Sørensen similarity index at both local and regional levels.

## Results

Sampling areas were selected in both counties according to the pre-determined criteria, namely inhabited houses, the presence of horses and poultry and next to a forest patch. A total of 1802 adult mosquitoes belonging to 55 species were captured in all the adult traps, 1360 of which were captured using the EVS traps (Additional file 1: Tables S1 and S2). In terms of medically essential species captured, *Culex quinquefasciatus* (*n* = 514) was the most frequent adult mosquito species captured in both sampling localities (Additional file 1: Tables S1 and S2). In comparison, 29 species from 11 different genera from 11 different larval habitats were obtained in the manual collections (Additional file 1: Table S3).

### Mosquitoes captured

**Fig. 1:**
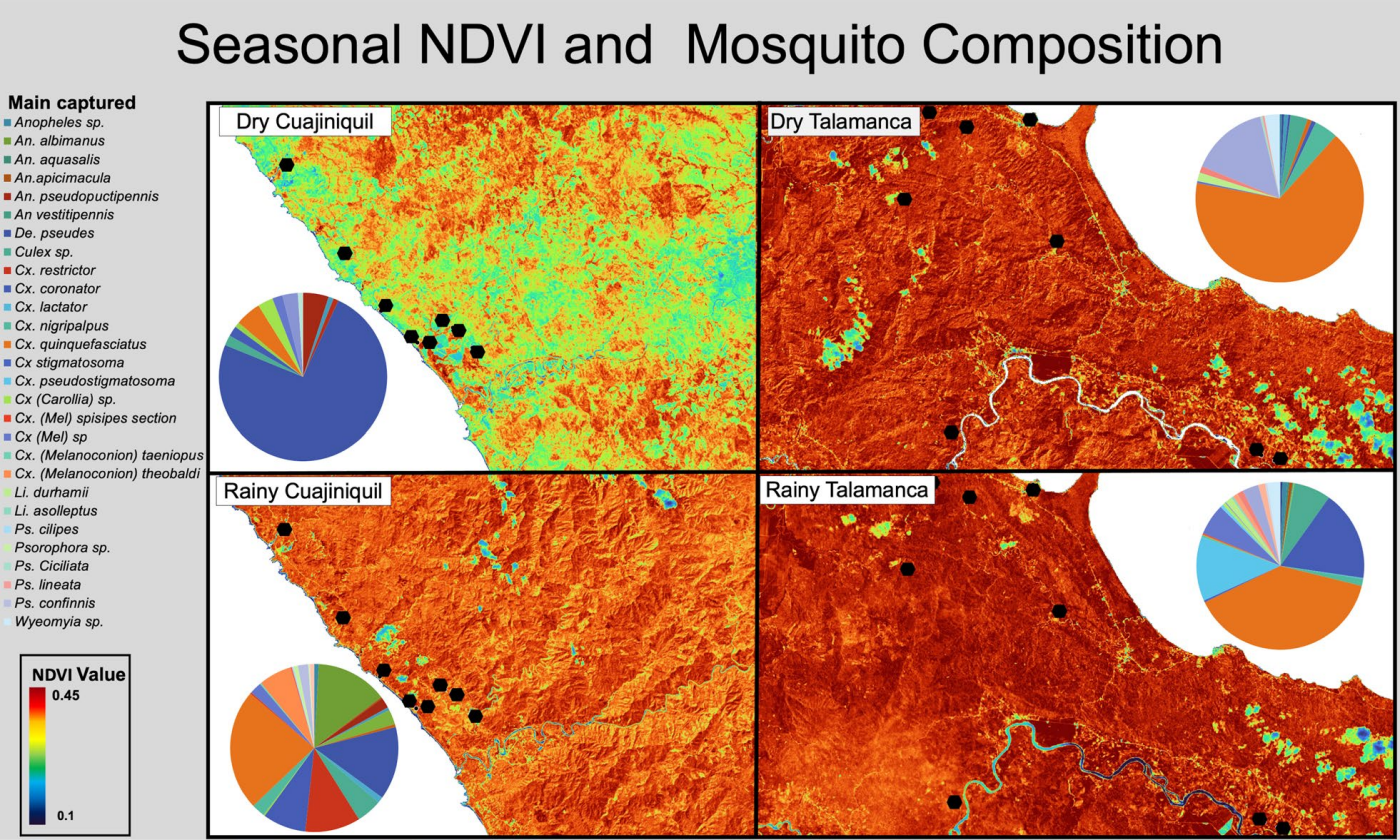
Variation in the Normalized Difference Vegetation Index (NDVI) and seasonal mosquito composition. Although the local and regional values of the NDVI were used for the statistical analysis, the complete Cuajiniquil (CU) and Talamanca (TA) datasets are shown in Table 2 to better illustrate the seasonal variation. Black hexagons indicate the sampling locations

The number of individual mosquitoes captured in the EVS traps in CU was much higher during the rainy season than during the dry season (*n* = 500 vs 101, respectively). For example, no *Anopheles albimanus* (*n* = 0) was caught during the dry season, and the number of *Cx. quinquefasciatus* captured decreased from 149 to five individuals between the rainy and dry seasons. *De. pseudes* (*n* = 75) was the only species for which the number of captures was not reduced during the dry season (Fig. 1). In contrast, the GTs was more productive during the dry season, with 64 captures during the dry season and five during the rainy season. The most common species in the GTs was also *Cx. quinquefasciatus* (*n* = 42). The OV traps were relatively unsuccessful in capturing mosquitoes during the dry season due to water evaporation and lack of rain, with only a few individuals of *Ae. aegypti* caught in one trap. Human-made habitats, such as rice plantations, were widely used by *An. albimanus*,* Culex coronator* and *Culex* (*Melanoconion*)* theobaldi* and, interestingly, this was the only habitat where *An. albimanus* larvae were found.

The most captured species in the EVS traps in TA were *Cx. quinquefasciatus* (*n* = 360) and *Cx. coronator* (*n* = 96). Medical important species belonging to the genus *Mansonia* (*n* = 66) were also captured in EVS traps in TA, as well as other species in lesser numbers (Additional file 1: Table S2). The low number of individuals of *Anopheles* spp. captured was unexpected. Similar results for the abundance of *Cx. quinquefasciatus* (*n* = 54) were obtained from the GTs. The only medically important species captured in the OVs was *Ae. aegypti*. Although the *Melanoconion* subgenus is of medical importance and *Culex* (*Melanoconion*)* psathaurus* was captured, its vector capacity has not been tested.

**Fig. 2:**
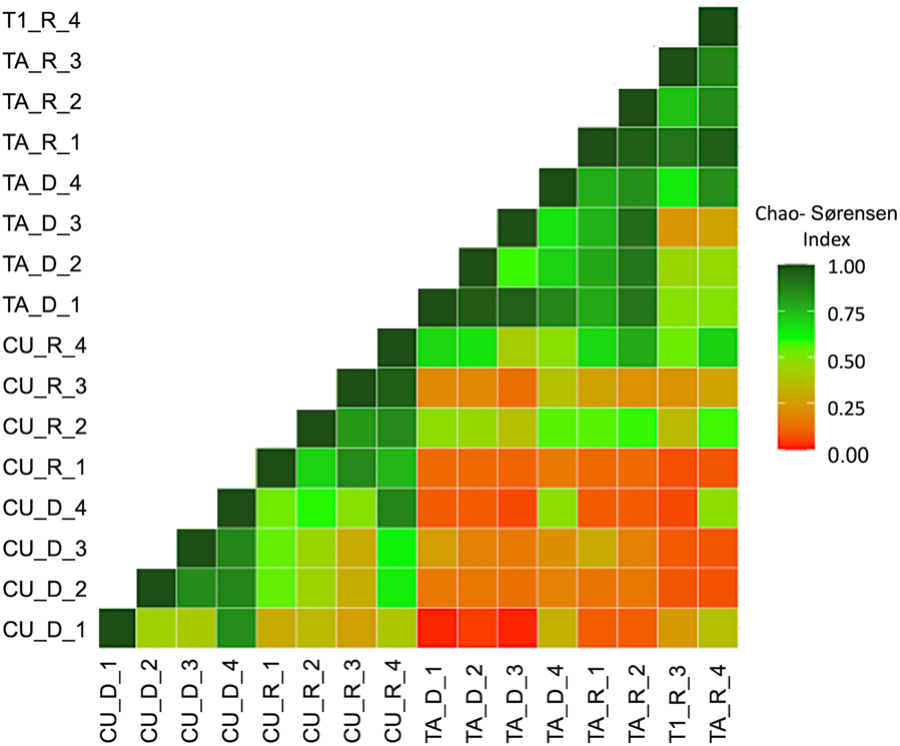
Species accumulation curve. The continuous line shows species accumulation per captured individual. The discontinuous line shows the extrapolation of species accumulated up to 1000 individuals. No more individuals were extrapolated due to the increase in estimation error

The species accumulation curve in both study sites shows that we did not attain the total sampling of all species (Fig. 2). Although the total number of individuals captured was higher in TA, the asymptote was clearer in CU, meaning that the species assemblies in CU were more completely sampled.

**Fig. 3:**
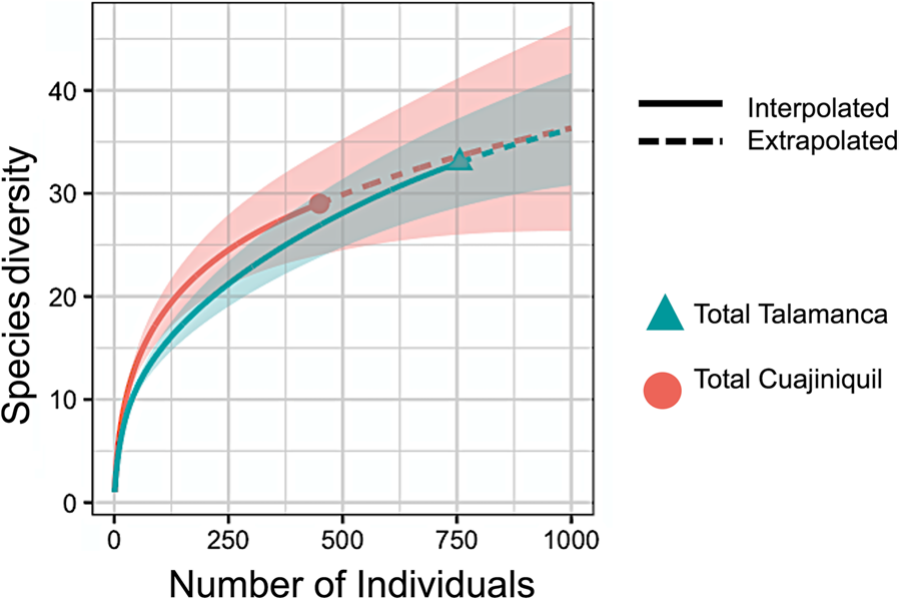
Chao-Sørensen similarity index. The similarity in species composition of each sampling area per season per county is shown. R, Rainy; D, dry; 1, domiciliary; 2, peridomiciliary; 3, animal pen; 4, forest

The most common habitats found in both sampling sites were identified as plastic containers, which were used by seven mosquito species. Other habitat types, such as Araceae plants and crab holes, were used only by single species, such as *Johnbelkinia leucopus* and *De. pseudes*, respectively. The most abundant species sampled were *Cx. coronator,* which was found in eight different habitat types, followed by *Limatus durhammii*, which was found in four habitat types. Other medically important species, such as *Ae. aegypti*, *Culex nigripalpus*, *Mansonia dyari*, *Haemagogus iridicolor* and *Haemagogus lucifer,* were also collected. In TA, *Cx. coronator* and *Ha. iridicolor* were collected from tree holes. Other mosquitoes, such as *Culex* (*Microculex*) spp., which only use phytotelmas as breeding sites [41], were also exclusively captured in OVs at TA. The species richness, Chao2 values and Shannon index are presented in Table 1. Chao2 values were higher for TA than for CU. The Chao-Sørensen index values for the EVS traps among the different areas and seasons are presented in Fig. 3, Additional file 1: Tables S4 and S5. The Chao-Sørensen value of each sampling point between seasons had a weak inverse correlation with the difference between the rainy and dry local NDVI (*R* = − 0.1) and NDWI (*R* = − 0.2) (Table 2). The regional NDVI per rainy and dry season was also inversely correlated with the Chao-Sørensen index among all of the diversity data per season per location (*R* = − 0.76) (Additional file 1: Table S5). The regional NDWI and Chao-Sørensen index correlation was *R* = − 0.61 (Table 2; Additional file 1: Table S6).

**Table 1:** Diversity and species richness estimations per county for each season and sampling area

Trap criteria	Setting	Species richness	Chao2 values for species richness (SD)	Shannon diversity index (*H′*)	Evenness
Rainy season	CU	25	36.63 (10.46)	2.45	0.71
	TA	27	52.19 (19.44)	2.06	0.56
Dry season	CU	12	13.94 (2.52)	1.14	0.46
	TA	26	22.75 (0.89)	1.29	0.47
Domiciliar	CU	16	36.63 (16.44)	2.13	0.77
	TA	19	26 (5.97)	1.11	0.38
Peridomiciliar	CU	15	17.81 (3.23)	2.32	0.86
	TA	14	18.36 (4.23)	1.04	0.39
Animal pen	CU	15	21.57 (6.07)	2.14	0.79
	TA	16	24.4 (7.15)	2.17	0.78
Forest	CU	20	40.63 (16.44)	1.98	0.66
	TA	23	65.47 (32.39)	2.31	0.74

*CU* Cuajiniquil,* SD* standard deviation,* TA *Talamanca

**Table 2:** Regional and local Normalized Difference Vegetation Index (NDVI) values per county and per season

Cuajiniquil			Talamanca		
	*Dry*	*Rainy*		*Dry*	*Rainy*
Regional NDVI	0.276	0.372	Regional NDVI	0.427	0.434
*Local NDVI (per house ID)*			*Local NDVI (per house ID)*		
CSCA	0.287	0.383	CTAA	0.432	0.447
CSCB	0.280	0.386	CTAB	0.429	0.450
CSCC	0.276	0.339	CTAC	0.443	0.424
CSCD	0.088	0.353	CTAD	0.419	0.452
CSCE	0.443	0.297	CTAE	0.437	0.452
CSCF	0.282	0.386	CTAF	0.430	0.406
CSCG	0.279	0.367	CTAG	0.423	0.426
CSCH	0.254	0.366	CTAH	0.407	0.412

Normalized Difference Vegetation Index (NDVI) and Normalized Difference Water Index (NDWI) were not calculated for sampling areas (DO, PE, PN, and FO) because the spatial resolution of the satellite images (30 ×30 m) did not allow their differentiation. House codes georeferences: CSCA: 9°59'35.95"N85°40'22.04"O; CSCB:10° 0'14.28"N85°41'7.77"O; CSCC:9°59'45.12"N85°41'58.87"O; CSCD:9°59'52.57"N85°41'44.99"O; CSCE:10° 0'40.80"N85°43'3.30"O; CSCF:10° 0'11.10"N85°40'56.41"O; CSCG:10° 2'22.44"N85°44'5.89"O; CSCH:10° 5'2.42"N85°46'6.88"O; CTAA:9°44'1.88"N82°52'21.43"O; CTAB:9°41'58.77"N82°54'21.03"O; CTAC:9°44'34.87"N82°53'39.20"O; CTAD:9°34'12.86"N82°43'30.70"O; CTAE:9°40'40.40"N82°49'53.14"O; CTAF:9°33'58.61"N82°55'23.06"O; CTAG: 9°34'22.62"N82°43'53.88"O; CTAH:9°43'52.98"N82°50'25.81"O

### Viral detection

RNA of ZIKV was detected in a pool of *Cx. quinquefasciatus* from CU, specifically from those mosquitoes captured in an EVS trap located in the FO setting in the rainy season. The sequence obtained matched a 2016 Colombian isolate (Accession number: MH179341.1) with 97.25% identity in BLAST. RNA of DENV type 3 was identified in a blood-fed *Cx. quinquefasciatus* collected in a GT located in a PE setting from TA during the rainy season. The sequence showed 96.7% similarity with a 2015 Colombian isolate (Accession number: MH544650.1).

### Blood meals

**Fig. 4:**
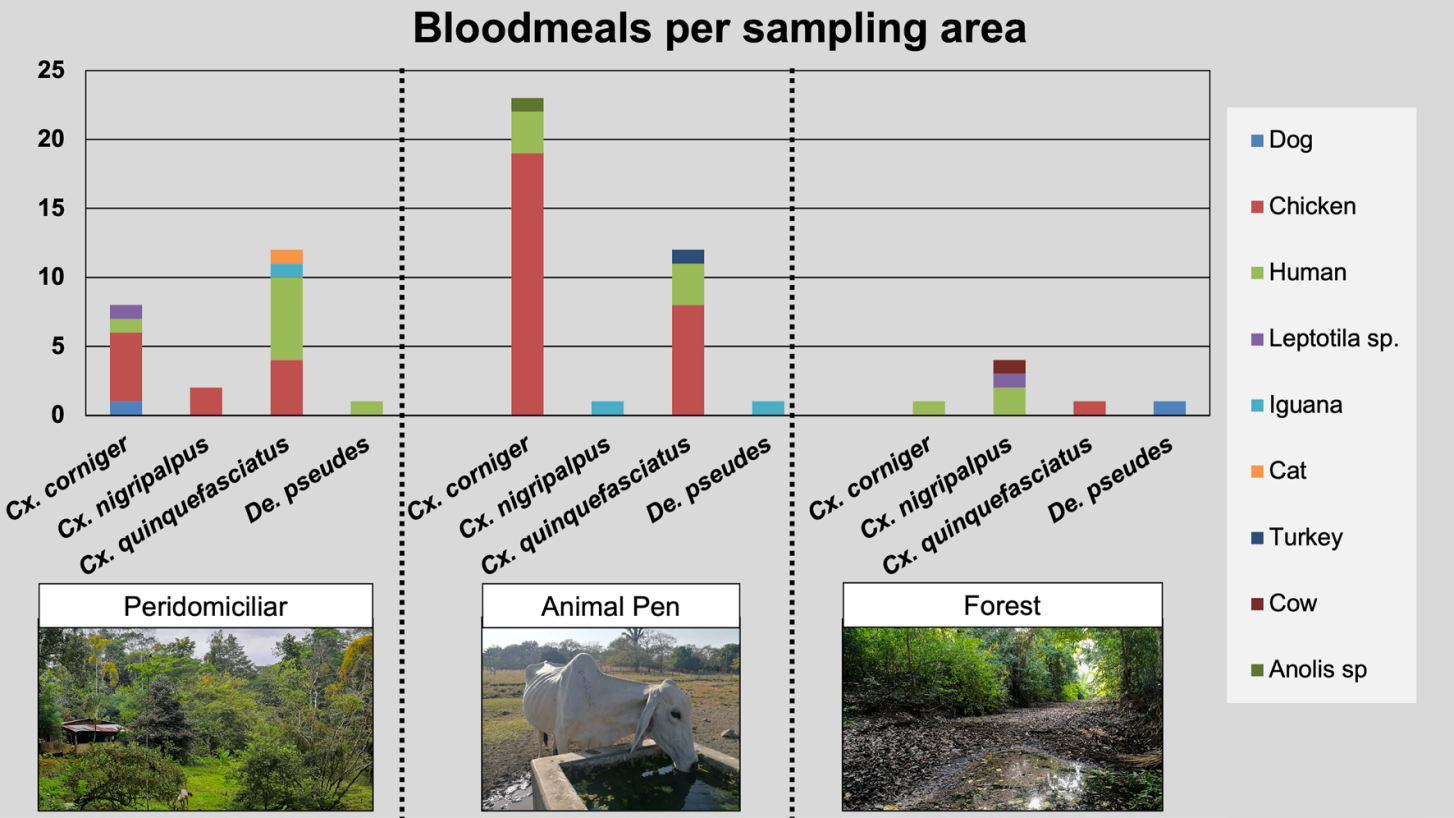
Blood-meal preferences. Blood-meal sources are plotted for each mosquito species in each sampling area

The blood meals identified in mosquitoes were mainly from domestic animals and humans. Most of the captured mosquitoes belonged to the genus *Culex* (*n* = 255), with *Cx. quinquefasciatus* (*n* = 126) and *Cx. corniger* (*n* = 92) being the most frequent species. Of these, 90 were gravid females, and 181 were blood-fed in the last 72 h. An attempt to obtain sequence data from blood in both gravid and blood-fed mosquitoes resulted in 65 identified blood meals. Overall, chicken was the main blood meal detected in engorged mosquitoes (39/65), most of which were collected in FE settings, followed by human blood (18/65). Of note, chicken blood was dominant in mosquitoes from the PE and PN settings, but it was not detected in those from the FO. Other blood-meal sources are detailed in Fig. 4.

## Discussion

Our results show that mosquito populations in CU and TA form complex communities that can change between seasons. The highest species richness estimations were obtained in TA in the rainy season. During this period, more habitats may be available for larval development due to rainfall, not only in artificial containers but also in phytotelma. Although the sampling effort in this study was limited, most of the species present in each area were captured based on the rarefaction curves (Fig. 2) and Chao2 values. The high standard deviation in the Chao2 value for TA (Table 1) plus the incapacity of reaching a curve asymptote can be explained by the number of rare species and/or singletons present in TA (Additional file 1: Table S2). This result is similar to those of other studies with comparable sampling efforts [42]. In this regard, sampling arthropods in tropical areas usually requires a relatively higher intensity compared to typical sampling efforts for other taxa [43].

Mosquito diversity (Shannon index) was higher in CU, although the species estimation was higher in TA, which suggests that CU populations are more homogeneous or contain fewer dominant species. The lower diversity values (high regularity) in TA may be due to the dominance of *Cx. quinquefasciatus*, which accounted for 47.6% (360/756) of the total captured mosquitoes in our samples (Fig. 1). Interestingly, the FO had the highest diversity when the Shannon index (H’) of TA values was compared between sampling settings. This can be further related to the anthropogenic pressure at the DO, PE and PN settings because of the affinity of *Cx. quinquefasciatus* to human activity, domestic animals and altered areas in the tropics [44]. Low diversity has been extensively associated as a risk factor of vector-borne diseases [45], and functional diversity has been established as a good predictor for higher R_0_ in vector-borne conditions [47]. Deforestation and changes in land use, such as cattle ranching, which reduce local diversity, have been proposed as risk factors for vector-borne and emerging infectious diseases [46]. Furthermore, recently productive landscapes, such as oil palm and pineapple plantations, have been associated with a higher presence of disease vectors [47].

Changes in species composition (Chao-Sørensen similarity index) was different among the sampling locations; nonetheless, their degree of variation between seasons differed for each county. Values for TA were nested within seasons, showing small changes in their species composition. In contrast, CU had a more dissimilar population between seasons, with a high turnover in the presence of medically important species, such as *An. albimanus* and *De. pseudes.* In this context, *Cx. quinquefasciatus* is a proven vector for several zoonotic arboviruses, including WNV and SLEV [48]. This variation in species composition can explain the high seasonality of VEEV and WNV observed in the CU area, where neurological disease due to arboviruses has a higher incidence during the rainy season [27].

The evenness in CU indicates that some species are dominant in this community year-long (e.g. *De. pseudes*). It has been proposed that the Shannon evenness index is a strong predictor of disease risk in multiple host communities [49]. Historically, most cases of WVN and VEEV have been recorded in the northern Pacific region of Costa Rica [50]. Therefore, vector community structure might play a fundamental role in viral activity based on the high variation in the diversity index and species richness.

The variation in seasonal NDVI, which is associated with the forest phenology, was higher for both regional and local values (CU standard deviation [SD]: 0.08; TA SD: 0.01) in CU, where the forest is deciduous during the dry season, giving a higher variation in NDVI [51, 52]. In contrast, the original forest in TA is a weakly seasonal forest (evergreen) and, therefore, great NDVI fluctuations are not expected [53]. At the local scale, buffered areas did not show a strong correlation (*R* = − 0.20) when compared with Chao-Sørensen values of the same sampling points between seasons. In contrast, the correlation of regional mean NDVI (all buffered areas per season) shows a stronger negative correlation (*R* = − 0.76) with the absolute difference in the NDVI values. The regional Chao-Sørensen index can represent a more representative change in the overall community since it considers more subsets of the total population, giving a more robust correlation when compared with the regional NDVI. However, the NDVI has been broadly used to predict population changes in different environments [54]. Arboviral encephalitis cases in horses have a high incidence during the rainy season in the region [27, 55]. In CU and its surroundings, species turnover and its relation with the NDVI could be an essential predictor of vector activity; furthermore, climate change and the El Niño-Southern Oscillation (ENSO) can extend rainy seasons in the tropics, consequently extending the period of vector patterns and regional viral activity [56–58]. Regarding NDWI, the correlation between the NDWI absolute difference between seasons (*R* = − 0.61) was weaker than the correlation with the NDVI. Nonetheless, the NDWI has also been proven to predict mosquito abundance in swimming pools and with longer mosquito seasons [59, 60].

These variations in the NDVI and NDWI were also reflected in the larval abundance between seasons in CU, where several of the OVs placed were completely dry upon later evaluation during the dry season (Additional file 1: Table S8). The absence of water-filled tree-holes limits the availability of suitable larval habitats for species such as *Haemagogus* spp. and *Sabethes* spp., which can be vectors of YFV and Mayaro. Similarly, in CU, rice fields were only present during the rainy season [61, 62]. Rice fields have been proven to be of public health importance in other countries for *An. albimanus* [63]. The absence/presence of an adult mosquito species is strongly related to the availability of appropriate habitats, which in the case of *An. albimanus* are rice fields [61, 62]; In CU, the drought in the dry season results in no available water in rice fields and causes the *An. albimanus* population to drop off almost completely. Currently, there is no active transmission of *Plasmodium* spp. in CU, although it is present in other areas of Costa Rica due to human movement and anthropogenic landscape changes, including illegal gold mining [64]. Therefore, anopheline larval habitat conditions and adult mosquito abundance at this site represent a potential risk for *Plasmodium* transmission in CU during the wet seasons. Moreover, the severe dry season can also influence arboviral incidence in the region, considering that some mosquito populations (e.g. *Anopheles quadrimaculatus*) can increase drastically after severe drought [65]. Although no precipitation data were analyzed, NDVI values reflect an increase/decrease in rainfall precipitations [66].

In contrast, species like *De. pseudes* have a similar abundance year-long. As this species breeds in salty water-filled crab holes, their larval habitat does not depend on rainfall and is, therefore, present year-round*. Deinocerites pseudes* is a proven vector of VEEV, so the continuous presence of this species can help maintain enzootic VEEV transmission in the reservoir host population.

The dry and rainy seasons of CU are highly different because of the excessive difference in rainfall during these seasons [67]. This difference can also reduce the population of tree-hole breeders during the dry period. Previously, OVs have been used in the tropical rainforest for sampling sylvatic enzootic vectors [29], but none have been used for sampling Culicidae in a tropical dry forest. Nonetheless, several studies in urban areas adjacent to tropical dry forests have shown a significant decrease in ovitrap capture success since human activities nearby can help maintain artificial containers filled with water [68, 69].

Most engorged females belonged to the *Culex* genus and were caught in the animal pen (Fig. 4 and Additional file 1: Table S9). *Culex* mosquitoes have a wide range of feeding hosts, including humans, domestic species and wildlife. Nonetheless, mosquito-feeding behavior can be aggregated, adapting to the available hosts [70]. Since our sampling was done in areas with a high presence of humans and domestic animals, these are expected to be the main feeding hosts. In addition, blood from a White-tipped dove (*Leptotila verreauxi)* was also detected, which is of interest given that neutralizing antibodies for WNV and SLEV have been detected before in this species [71]. Although this dove plays a potential role in the epidemiology of some arboviruses, the importance of these findings is that vector species, such as *Cx. quinquefaciatus* and *De. pseudes*, are feeding on putative enzootic hosts and dead-end hosts, which is necessary for viral transmission to horse and human populations. The implications of reptile blood in terms of transmission are probably less important than those of avian blood, although some species can serve as amplifiers for WNV [72].

The frequent detection of chicken blood in the mosquitoes collected at both sites can have repercussions on the epidemiological cycle. Chickens are refractory to WNV [73] infections and work as sentinels for WNV [74]. This species has been proven to work as a zoo-prophylactic species for other vector-borne diseases [75–77]. The possibility that chickens might be taking a zoo-prophylactic role in arborvirus transmission in Costa Rica needs to be further explored since chickens are common backyard animals in rural areas. In contrast to other countries, cases of WNV in Costa Rica are rare in humans and horses [27, 78]. The most prevalent arbovirus in the country with a confirmed enzootic cycle is VEEV [79], but its prevalence is still infrequent compared with other arboviruses, such as DENV.

The detection of DENV and ZIKV RNA was not unexpected in these areas since both viruses are prevalent in Costa Rica. DENV type 3 has not been detected in humans in Costa Rica since 2016 [80]. Although the amplified region of the cDNA is short, this positive sample had a 96.7% similarity with a 2016 Colombian isolate (MH544650.1). Considering that this DENV type 3-positive sample was from a *Cx quinquefasciatus* that contained human blood, it is likely that the viral RNA was from a viremic human since *Cx. quinquefasciatus* is not epidemiologically relevant as a DENV vector [81].

Regarding ZIKV, this species has only been proven to be an efficient vector in a few vector competence studies, but the consensus is that it is not a primary ZIKV vector [82]. We do not consider that *Cx. quinquefasciatus* has a significant role in DENV or ZIKV transmission to humans. Overall, viral detection was unexpectedly low. Arboviruses usually circulate at a low prevalence between vectors [83]. Although our rarefaction curves indicate that we sampled most of the species in the area, the vector population captured may be considered to be low (e.g. *Cx. quinquefasciatus*: 514/1360).

## Conclusions

Despite their geographical closeness, CU and TA districts have different seasonal dynamics and population turnover. These factors can be important in further prediction and ecological modeling for arboviruses in Costa Rica and other neotropical countries that share tropical rain and dry forests. In addition, the NDVI can have more influence on mosquito diversity on a regional scale than on a local scale. However, year-long continuous sampling is required to understand local dynamics better. This can further relate to how anthropogenic pressure (deforestation, changes in land use) can affect the mosquito vectors present in the area. Since mosquito feeding preferences are strongly guided by host availability, these changes in land use and resource availability can modify the community of putative vectors in an epidemiological context.

## Supplementary Information

**Additional file 1: Table S1.** Mosquitoes captured with EVS traps in Cuajiniquil. DO: Domiciliary, PE: Peridomiciliary, PN: Animal Pen, FO: Forest. ** Table S2.** Mosquitoes captured with EVS traps in Talamanca. DO: Domiciliary, PE: Peridomiciliary, PN: Animal Pen, FO: Forest.** Table S3.** Mosquito larvae captured with manual collection. ** Table S4. **Regional Chao- Sørensen Index.** Table S5.** Chao- Sørensen Similarity Index. ** Table S6. **Chao- Sørensen Index and NDVI . ** Table S7.** Chao- Sørensen Index and NDWI. ** Table S8.** Mosquito larvae captured with ovitraps. ** Table S9.** Mosquitoes captured with GT traps.

## Data Availability

The data are available for any interested upon request to the corresponding author.

## Abbreviations

CU: Cuajiniquil
DENV: Dengue virus
DO: Domiciliary
EEEV: Eastern equine encephalitis virus
ENSO: El Niño-Southern Oscillation
EVS: Encephalitis vector surveillance (traps)
FO: Forest
GT: CDC female gravid traps
NDVI: Normalized Difference Vegetation Index
OV: Ovitraps
PE: Peridomiciliary
PN: Animal pen
SLEV: Saint Louis encephalitis virus
TA: Talamanca
VEEV: Venezuelan equine encephalitis virus
WNV: West Nile virus
YFV: Yellow fever virus
ZIKV: Zika virus
