# Supplementary material for: Seasonal changes in the diversity, host preferences and infectivity of mosquitoes in two arbovirus-endemic regions of Costa Rica
Source: Parasit Vectors. 2023 Jan 26;16:34. doi: 10.1186/s13071-022-05579-y (PMC9881273; doi:10.1186/s13071-022-05579-y)
Supplement: Supplementary file 1 — Additional file 1: Table S1. Mosquitoes captured with EVS traps in Cuajiniquil. DO: Domiciliary, PE: Peridomiciliary, PN: Animal Pen, FO: Forest. Table S2. Mosquitoes captured with EVS traps in Talamanca. DO: Domiciliary, PE: Peridomiciliary, PN: Animal Pen, FO: Forest. Table S3. Mosquito larvae captured with manual collection. Table S4. Regional Chao- Sørensen Index. Table S5. Chao- Sørensen Similarity Index. Table S6. Chao- Sørensen Index and NDVI . Table S7. Chao- Sørensen Index and NDWI. Table S8. Mosquito larvae captured with ovitraps. Table S9. Mosquitoes captured with GT traps. [file 13071_2022_5579_MOESM1_ESM.pdf]

This file contains the tables detailing the number of individuals capture in each trap type specie per county per season. Also details the species of larvae captured by manual collection and the breeding habitat that they were found.

Table S1. Mosquitoes captured with EVS traps in Cuajiniquil. DO: Domiciliary, PE: Peridomiciliary, PN: Animal Pen, FO: Forest.

Mosquito Species	Season										Total
	Rainy					Dry					
	DO	PE	PN	FO	Total	DO	PE	PN	FO	Total	
Anopheles sp.	0	0	3	0	3	0	0	0	0	0	3
An. albimanus	1	21	9	18	49	0	0	0	0	0	49
An.apicimacula	0	1	0	0	1	0	0	0	0	0	1
An. pseudopuctipennis	4	0	3	5	12	0	0	0	5	5	17
An. neomaculipalpus	1	0	1	1	3	0	0	1	0	1	4
An. punctimacula	3	5	1	4	13	0	0	0	0	0	13
Ae. squamipennis	0	2	0	0	2	0	0	0	0	0	2
De. costaricensis	0	0	0	0	0	0	1	0	0	1	1
De. pseudes	7	25	9	15	56	2	10	14	49	75	131
De. epitedeus	0	4	0	0	4	0	0	0	0	0	4
Culex sp.	13	0	2	16	31	1	0	0	1	2	33
Cx. restrictor	15	4	9	24	52	0	0	0	0	0	52
Cx. coronator	23	0	5	24	52	1	0	0	1	2	54
Cx. chiresteri	0	1	0	0	1	0	0	1	0	1	2
Cx. nigripalpus	0	0	8	1	9	0	0	0	0	0	9
Cx. quinquefasciatus	63	5	12	69	149	0	1	2	2	5	154
Cx. mollis	1	0	0	1	2	0	0	0	0	0	2
Cx (Carollia) sp.	0	0	0	0	0	2	0	0	1	3	3
Cx (Meanoconion) sp.	2	1	2	5	10	0	0	0	2	2	12
Cx. (Mel.) taeniopus	1	0	0	1	2	0	0	0	0	0	2
Cx. (Mel.) theobaldi	4	8	0	16	28	0	0	0	0	0	28
Cx. (Mel.) erraticus	1	0	0	1	2	0	0	0	0	0	2
Haemagogus sp.	0	0	0	0	0	0	0	2	1	3	3
Psorophora sp.	0	1	1	1	3	0	0	0	0	0	3
Ps. cilipes	1	0	0	1	2	0	0	0	0	0	2
Ps. confinnis	1	2	4	1	8	0	0	0	0	0	8
Sabethes undosus	1	0	0	1	2	0	0	0	0	0	2
Tricoprosopon digitatum	0	0	0	0	0	1	0	0	0	1	1
Uranotaenia coatzalcoalcos	1	0	2	1	4	0	0	0	0	0	4
**Total**	143	80	71	206	500	7	12	20	62	101	601

Table S2. Mosquitoes captured with EVS traps in Talamanca. DO: Domiciliary, PE: Peridomiciliary, PN: Animal Pen, FO: Forest.

Mosquito Species	Season										Total
	Rainy					Dry					
	ID	PE	ES	BO	Total	ID	PE	ES	BO	Total	
Ae. aegypti	0	0	2	0	2	0	0	0	0	0	2
Anopheles sp.	1	1	0	3	5	0	0	1	0	1	6
An. aquasalis	0	0	2	0	2	0	0	0	0	0	2
An. apicimacula	0	0	0	3	3	0	0	0	0	0	3
An. neivai	0	0	0	0	0	0	0	0	1	1	1
An. neomaculipalpus	0	0	0	0	0	1	0	0	1	2	2
An. vestitipennis	0	0	1	0	1	0	0	0	0	0	1
De. pseudes	0	0	0	0	0	0	0	0	1	1	1
Co. nigricans	1	0	0	0	1	0	0	0	0	0	1
Culex sp.	4	2	3	28	37	1	1	0	6	8	45
Cx. declarator	0	0	0	0	0	1	0	0	1	2	2
Cx. coronator	5	4	8	73	90	0	0	0	2	2	92
Cx. lactator	1	0	0	0	1	0	0	0	0	0	1
Cx. nigripalpus	1	1	0	5	7	1	1	3	9	14	21
Cx. quinquefasciatus	95	89	4	13	201	104	34	18	3	159	360
Cx. stigmatosoma	0	2	0	0	2	0	0	0	0	0	2
Cx. pseudostigmatosoma	40	1	11	12	64	0	0	0	0	0	64
Cx. pleuristratus	0	0	2	0	2	0	0	0	0	0	2
Cx. secundus	0	0	0	1	1	0	0	0	0	0	1
Cx. (Mel.) spisipes section	0	1	0	0	1	0	0	0	0	0	1
Cx. (Mel) sp.	2	1	7	27	37	0	0	0	1	1	38
Haemagogus sp.	1	0	0	0	1	0	0	0	0	0	1
Limatus durhamii	1	1	1	4	7	0	1	0	3	4	11
Li. asolleptus	0	0	0	2	2	0	0	0	0	0	2
Mansonia sp.	1	0	0	4	5	0	0	0	0	0	5
Ma. dyari	1	0	2	3	6	1	2	0	0	3	9
Ma. titillans	2	1	3	10	16	1	0	25	10	36	52
Psorophora. ciciliata	0	0	0	0	0	0	0	0	1	1	1
Ps. ferox	2	2	1	2	7	0	0	0	0	0	7
Ps. lineata	0	0	0	0	0	1	0	0	0	1	1
Shannonia fluvitialis	0	0	0	1	1	0	0	0	0	0	1
Wyeomyia sp.	2	0	5	6	13	3	1	0	3	7	20
Wy. complosa	0	0	0	1	1	0	0	0	0	0	1
**Total**	160	106	52	198	516	114	40	47	42	243	759

Table S3. Mosquito larvae captured with manual collection.

Breeding Site	County	Capture Species
Araceae	Talamanca	*Johnbelkinia leucopus*
Rice fields	Cuajiniquil	*Anopheles albimanus*
		Culex coronator
		Culex theobaldi
Bamboo	Talamanca	*Haemagogus lucifer*
Cattle waterer	Cuajiniquil	*Culex quinquefasciatus*
		Culex coronator
Bromelid	Cuajiniquil	*Wyeomyia abebela*
	Talamanca	*Culex pleuristriatus*
		Culex rejector
		Toxorhynchites superbus
Puddle	Talamanca	*Culex coronator*
		Culex nigripalpus
		Culex lactator
Coconut shells	Talamanca	*Limatus durhamii*
Dumped plastic containers	Cuajiniquil	*Aedes aegypti*
		Aedes septemstriatus
		Culex quinquefasciatus
		Culex mollis
		Culex corniger
	Talamanca	*Culex coronator*
		Limatus durhamii
Pond	Talamanca	*Culex chidesteri*
		Mansonia dyari
Tire	Cuajiniquil	*Culex coronator*
	Talamanca	*Aedes aegypti*
		Culex coronator
		Limatus durhamii
Tree Hole	Talamanca	*Culex coronator*
		Haemagogus iridicolor
Crab Hole	Cuajiniquil	*Deinocerites pseudes*
Swamp	Talamanca	*Culex aquarius*
		Culex conservator
		Uranotaenia coatzacoalcos
Seasonal pond	Cuajiniquil	*Culex mutator*
		Culex (Melanoconion) sp.
		Culex conspirator
		Uranotaenia lowii
		Uranotaenia coatzacoalcos

Table S4. Regional Chao- Sørensen Index. Similarity index was calculated using the EVS traps data. CU= Cuajiniquil, TA= Talamanca, R= Rainy season, D= Dry season.

Site 1	Site 2	Chao-Sorensen-Similarity Index
CU_R	CU_D	0,694
CU_R	TA_R	0,536
CU_R	TA_D	0,653
CU_D	TA_D	0,212
CU_D	TA_D	0,795
TA_R	TA_D	0,876

Table S5. Chao- Sørensen Similarity Index. Similarity on species composition of each sampling area per season per county. CU: Cuajiniquil, TA: Talamanca, R: Rainy, D: Dry, 1: Domiciliary, 2: Peridomiciliary, 3:Animal Pen, 4:Forest.

	CU_D_1	CU_D_2	CU_D_3	CU_D_4	CU_R_1	CU_R_2	CU_R_3	CU_R_4	TA_D_1	TA_D_2	TA_D_3	TA_D_4	TA_R_1	TA_R_2	TA_R_3	TA_R_4
CU_D_1	1	0.426	0.406	0.848	0.302	0.352	0.264	0.394	0.017	0.043	0	0.323	0.094	0.094	0.243	0.366
CU_D_2	0.426	1	0.854	0.867	0.352	0.436	0.311	0.638	0.153	0.152	0.137	0.18	0.146	0.152	0.08	0.073
CU_D_3	0.406	0.854	1	0.868	0.264	0.445	0.304	0.627	0.258	0.179	0.159	0.218	0.3	0.179	0.087	0.079
CU_D_4	0.848	0.867	0.868	1	0.394	0.592	0.49	0.866	0.092	0.092	0.06	0.476	0.193	0.12	0.158	0.17
CU_R_1	0.302	0.542	0.542	0.524	1	0.696	0.858	0.754	0.12	0.119	0.11	0.159	0.116	0.119	0.07	0.079
CU_R_2	0.352	0.436	0.445	0.592	0.696	1	0.818	0.856	0.474	0.452	0.374	0.554	0.556	0.584	0.352	0.577
CU_R_3	0.264	0.311	0.304	0.49	0.858	0.818	1	0.956	0.196	0.195	0.132	0.373	0.273	0.22	0.228	0.277
CU_R_4	0.394	0.638	0.627	0.866	0.754	0.856	0.956	1	0.682	0.661	0.414	0.48	0.68	0.781	0.529	0.699
TA_D_1	0.017	0.153	0.258	0.092	0.12	0.474	0.196	0.682	1	0.97	0.953	0.866	0.784	0.907	0.488	0.491
TA_D_2	0.043	0.152	0.179	0.092	0.119	0.452	0.195	0.661	0.97	1	0.581	0.697	0.788	0.901	0.445	0.459
TA_D_3	0	0.137	0.159	0.06	0.11	0.374	0.132	0.414	0.953	0.581	1	0.662	0.764	0.929	0.235	0.271
TA_D_4	0.323	0.18	0.218	0.476	0.159	0.554	0.373	0.48	0.866	0.697	0.662	1	0.774	0.843	0.644	0.846
TA_R_1	0.094	0.146	0.3	0.092	0.116	0.556	0.273	0.68	0.784	0.788	0.764	0.774	1	0.961	0.909	0.962
TA_R_2	0.094	0.152	0.179	0.092	0.119	0.584	0.22	0.781	0.907	0.901	0.929	0.843	0.961	1	0.731	0.855
TA_R_3	0.243	0.08	0.087	0.06	0.07	0.352	0.228	0.529	0.488	0.445	0.235	0.644	0.909	0.731	1	0.872
TA_R_4	0.366	0.073	0.079	0.476	0.079	0.577	0.277	0.699	0.491	0.459	0.271	0.846	0.962	0.855	0.872	1

Table S6. Chao- Sørensen Index and NDVI . Correlation between Chao- Sørensen Index and NDVI absolute difference between seasons. R= -0.768. CU= Cuajiniquil, TA= Talamanca, R= Rainy, D=Dry.

First Sample	Second Sample	Chao-Sorensen-Index	Absolute difference NDVI
CU_R	CU_D	0,694	0,096
CU_R	TA_R	0,536	0,061
CU_R	TA_D	0,653	0,054
CU_D	TA_R	0,212	0,157
CU_D	TA_D	0,795	0,096
TA_R	TA_D	0,876	0,007

Table S7. Chao- Sørensen Index and NDWI . Correlation between Chao- Sørensen Index and NDVI absolute difference between seasons. R= -0,619. CU= Cuajiniquil, TA= Talamanca, R= Rainy, D=Dry.

First Sample	Second Sample	Chao-Sorensen-Index	Absolute difference NDWI
CU_LL	CU_SE	0,694	0,077
CU_LL	TA_LL	0,536	0,016
CU_LL	TA_SE	0,653	0,043
CU_SE	TA_LL	0,212	0,093
Cu_SE	TA_SE	0,795	0,027
TA_LL	TA_SE	0,876	0,027

Table S8. Mosquito larvae captured with ovitraps. PE: Peridomiciliary, PN: Animal Pen, FO: Forest.

Mosquito Species	Cuajiniquil						Talamanca						Total
	Rainy			Dry			Rainy			Dry			
	PE	PN	FO	PE	PN	FO	PE	PB	FO	PE	PN	FO	
Aedes aegypti	0	1	0	0	10	0	2	0	2	0	0	0	15
Aedes albopictus	0	1	0	0	0	0	0	0	0	0	0	0	1
Aedes septemstriatus	0	0	5	0	0	0	0	0	0	0	0	0	5
Aedes epactius	0	2	0	0	0	0	0	0	0	0	0	0	2
Culex (Mel.) psatharus	0	0	0	0	0	0	0	0	0	0	0	3	3
Limatus durhamii	1	4	6	0	0	0	39	2	17	1	23	0	93
Limatus asolleptus	0	0	0	0	0	0	5	5	20	0	0	0	30
Toxorhynchites hypoptes	0	0	0	0	0	0	0	0	0	0	0	1	1
Toxorhynchites moctezuma	0	1	0	0	0	0	0	0	1	0	0	0	2
Wyeomyia aporomona	0	0	0	0	0	0	0	0	0	0	0	1	1
**Total**	1	9	11	0	10	0	46	7	40	1	23	5	153

Table S9. Mosquitoes captured with GT traps. PE: Peridomiciliary, PN: Animal Pen, FO: Forest.

Mosquito species	Cuajiniquil						Talamanca						Total
	Rainy			Dry			Rainy			Dry			
	PE	PN	FO	PE	PN	FO	PE	PN	FO	PE	PN	FO	
Anopheles apicimacula	0	0	0	0	0	0	0	1	0	0	0	0	1
Anopheles pseudopunctipennis	0	0	0	0	0	1	0	0	0	0	0	0	1
Aedes aegypti	0	0	0	0	1	0	0	0	0	0	1	0	2
Culex sp.	1	0	0	1	1	2	5	0	0	1	0	0	11
Culex corniger	0	0	0	0	0	0	18	55	4	7	7	1	92
Culex coronator	0	0	0	0	0	0	1	0	2	0	0	0	3
Culex nigripalpus	2	1	0	4	0	5	0	2	2	1	0	0	17
Culex quinquefasciatus	0	0	0	18	13	11	33	20	1	19	11	0	126
Culex pseudostigmatosoma	0	0	0	0	0	0	0	0	2	0	0	0	2
Culex restrictor	0	0	0	0	1	0	0	0	0	0	0	0	1
Culex restuans	0	0	0	0	0	0	0	0	0	0	0	0	0
Deinocerites pseudes	0	0	0	0	3	2	0	0	0	0	0	0	5
Haemagogus iridicolor	0	0	0	0	1	0	0	0	0	0	0	0	1
Limatus durhamii	0	0	0	0	0	0	0	1	1	0	0	0	2
Psorophora confinnis	0	0	1	0	0	0	0	0	0	0	0	0	1
Uranotaenialowii	0	0	2	0	0	0	0	1	4	0	2	0	9
**Total**	3	1	3	23	20	21	57	80	16	28	21	1	274
